# Work Stress Interventions in Hospital Care: Effectiveness of the *DISCovery* Method

**DOI:** 10.3390/ijerph15020332

**Published:** 2018-02-13

**Authors:** Irene Niks, Jan de Jonge, Josette Gevers, Irene Houtman

**Affiliations:** 1 Human Performance Management Group, Eindhoven University of Technology, P.O. Box 513, 5600 MB Eindhoven, The Netherlands; irene.niks@tno.nl (I.N.); j.m.p.gevers@tue.nl (J.G.); 2 The Netherlands Organization for Applied Scientific Research, TNO, P.O. Box 3005, 2301 DA Leiden, The Netherlands; irene.houtman@tno.nl; 3 School of Psychology, Asia Pacific Centre for Work Health and Safety, University of South Australia, P.O. Box 2471, Adelaide, SA 5001, Australia

**Keywords:** work stress, job demands, job resources, off-job recovery, interventions, DISC-R Model, *DISCovery* method, multiple-case study, health care workers

## Abstract

Effective interventions to prevent work stress and to improve health, well-being, and performance of employees are of the utmost importance. This quasi-experimental intervention study presents a specific method for diagnosis of psychosocial risk factors at work and subsequent development and implementation of tailored work stress interventions, the so-called *DISCovery* method. This method aims at improving employee health, well-being, and performance by optimizing the balance between job demands, job resources, and recovery from work. The aim of the study is to quantitatively assess the effectiveness of the *DISCovery* method in hospital care. Specifically, we used a three-wave longitudinal, quasi-experimental multiple-case study approach with intervention and comparison groups in health care work. Positive changes were found for members of the intervention groups, relative to members of the corresponding comparison groups, with respect to targeted work-related characteristics and targeted health, well-being, and performance outcomes. Overall, results lend support for the effectiveness of the *DISCovery* method in hospital care.

## 1. Introduction

Health care staff is widely regarded as a group that is at high risk of work stress and job dissatisfaction [1,2]. High levels of work stress are related to higher sickness absenteeism rates [3] and decreased performance [4], thereby jeopardizing patient safety [3]. Hence, effective interventions to prevent work stress and to improve health, well-being, and performance of health care employees are badly needed [5].

Although stress prevention has received considerable attention over the last two decades, there is still a gap between theoretical knowledge regarding work stress prevention and corresponding practical applications [5,6]. Scientifically well-performed studies on work stress and performance interventions are still scarce, and organization-level interventions often fail to achieve the desired results [7,8,9,10]. Kompier and Kristensen [9] argued that the majority of work stress management programs have a “one size (or one pill) fits all” character (p. 170) with some interventions resembling “smoking cessation courses for non-smokers” (p. 182). In other words, there is a lack of proper diagnosis of risk factors at work (i.e., job stressors) and risk groups.

The current intervention study presents a specific method for diagnosis of risk factors at work and subsequent development and implementation of tailored work stress and performance interventions, the so-called *DISCovery* method [11]. Accordingly, the aim of this study is to quantitatively assess the effectiveness of the *DISCovery* method in hospital care. Following the recommendations of Kompier and Kristensen [9], we use a multiple-case study approach, in which the context is different for each of the cases. This provides the opportunity to explore holistic explanations within and across settings, taking into account the dynamic and process nature of unfolding events that are embedded in an organizational context [12]. As such, this intervention study contributes to bridging the gap between theory and practice in work stress prevention.

The *DISCovery* method has been developed by De Jonge and colleagues [11] and is a method to improve employee health, well-being, and performance, through development and implementation of tailored work stress interventions that are based on a proper diagnosis of risk factors at work. Specifically, it is founded on key principles of the Demand-Induced Strain Compensation Recovery (DISC-R) Model [13,14,15] and participatory action research (PAR) [16]. We will address these principles consecutively.

The DISC-R Model is a work stress model that is used as a theoretical framework for the identification of risk factors and risk groups at work. The model comprises three central components: job demands, job resources, and the recovery concept of “detachment from work”. Job demands are defined as work-related tasks that require physical and/or psychological effort from the worker [17]. In other words, job demands place a certain amount of strain on employees. Job resources, on the other hand, are instrumental or psychological means at work that can be used to deal with job demands, such as job control and workplace social support [18]. As such, the use of job resources can counteract negative strain effects of job demands. Detachment from work is defined as an individual’s sense of being away from the work situation [19]. By detaching from work, functional bodily systems that were activated during work can return to baseline levels [15]. In general, it can be seen as a promising strategy to recover from work-related strain [20]. Thus, similar to job resources, detachment from work has a mitigating function with respect to detrimental effects of high job demands.

The *DISCovery* method is based on two main principles of the DISC-R Model [15]. First, the model proposes that a balance between job demands, job resources, and detachment from work will lead to favorable outcomes in terms of employee health, well-being and performance, whereas an imbalance will lead to unfavorable outcomes, such as job dissatisfaction or emotional exhaustion. Put differently, job demands can lead to negative strain effects unless employees (1) have sufficient job resources to deal with the demands and (2) can recover sufficiently from effort expenditure. Because job demands often cannot easily be reduced, the focus in this study is on combatting work stress by enhancing job resources and detachment instead. Second, in line with the DISC-R Model, stress-buffering effects of job resources and detachment from work are expected to be the strongest if they are specific and targeted, rather than broad and general [15]. Job demands, job resources, and detachment from work can each be divided into cognitive, emotional, and physical elements. Prior studies have shown empirical support for this assumption [15,21]. For example, health care employees often have to carry out complex tasks under time pressure (cognitive demands), deal with suffering patients (emotional demands), or lift heavy objects (physical demands). Similarly, examples of different types of job resources are decision authority (cognitive), emotional support from co-workers (emotional), and lifting devices (physical). With regard to detachment from work, one can direct one’s thoughts to a non-work topic (cognitive), put work-related emotions aside (emotional), or shake off physical exertion (physical). In sum, unlike other job stress models, the DISC-R Model incorporates both job resources and recovery from work as means to counterbalance high job demands. In addition, it offers specific guidelines about the kind of job resources and recovery that should be aimed for, by proposing that job resources and recovery that correspond with specific types of job demands are most effective (‘matching principle’). Applying these DISC-R propositions to real practice, we expect that interventions are most likely to be effective if they are tailored to specific job demands (i.e., cognitive, emotional, or physical) and particularly aimed at changing corresponding job resources and recovery aspects.

The approach towards development and implementation of the interventions in this study is based on principles of Participatory Action Research, or PAR [16]. The philosophy of PAR is that organizational interventions designed to promote employee health cannot take place without the participation and experience of the subjects under study. This is in line with Nielsen et al. [22], who stated that occupational health interventions have the best chance of achieving a significant impact if they follow a structured and participatory intervention process. Moreover, Dollard and colleagues [16] argued that PAR has the potential to contribute to organizational sustainability, as organization members learn to solve self-identified problems. Previous studies have shown the effectiveness of PAR approaches in intervention research e.g., [23].

The aim of the *DISCovery* method is optimizing the balance between job demands, job resources, and recovery from work through three successive steps, which will be discussed later in the method section. Using this method within a multiple-case study approach with intervention and comparison groups, we expect tailored work-oriented interventions to have positive effects on job resources and recovery from work, and on employee health, well-being, and performance outcomes for the intervention groups. In this particular context, a distinction can be made between two types of outcomes cf. [21]. Job resources and recovery from work can be viewed as more acute or proximal outcomes of the interventions, as these work-related characteristics are directly targeted by the interventions, and, therefore, expected to be most sensitive to the intervention process [24]. Improved employee health, well-being, and performance, on the other hand, are referred to as more chronic or distal outcomes, as it may take more time for such effects to unfold compared to proximal outcomes [25]. Hence, two main hypotheses guided our study:

**Hypothesis** **1** **(H1).** *Relative to members of the comparison groups, members of the intervention groups show positive changes in targeted work-related characteristics after intervention implementation (proximal outcomes).*

**Hypothesis** **2** **(H2).** *Relative to members of the comparison groups, members of the intervention groups show improvements in targeted employee health, well-being, and performance outcomes after intervention implementation (distal outcomes).*

Investigating aspects of unique, tailor-made intervention programs within multiple cases inherently yields an extensive amount of information. Throughout this paper we will, therefore, focus on the main points of the study and refer to appendices for more detailed information.

## 2. Materials and Methods

### 2.1. Study Design, Data, and Procedure

The research was conducted in a multi-located Dutch general hospital over the course of two years using a three-wave longitudinal, quasi-experimental, multiple-case study design. Initially, three existing organizational departments situated at two different locations agreed upon participation in the study: a nursing department (i.e., Case 1), a laboratory (i.e., Case 2), and an emergency room department (i.e., Case 3). At the start of the study, each department consisted of two or more separate work units at different locations, thereby allowing a subsequent division into intervention and comparison groups within each department. However, due to unforeseen organizational changes, the comparison group within the emergency room department merged with the intervention group a few months after baseline data collection (Time 1). Because no other suitable comparison group existed for this department, we decided to adjust the study design and to continue with two rather than three cases.

All employees within the two remaining departments (*N* = 111) were invited to participate on a voluntary basis. They received an email with a unique link to an online survey on three occasions: October 2011 (Time 1); January 2013 (Time 2); and November 2013 (Time 3). Intervention development and implementation started after Time 1. This time frame was based on the estimated time needed to complete the *DISCovery* method in this particular study. In addition, it allowed for the evaluation of intervention effects at two different time points (Time 2 and Time 3), as to investigate possible differences between proximal and distal outcomes of the interventions. Everyone in the sample was invited to fill out the surveys at Time 2 and Time 3, regardless of whether they had completed the survey at the previous time point. Table 1 shows more detailed information about the response rates for each subsample. Online surveys were linked to the employees’ email addresses for second-round and third-round identification. To guarantee confidentiality of the data, the identification information was only available to the researchers and exclusively used for data-management purposes. A medical ethics committee confirmed that the current study was carried out in accordance with the applicable rules concerning the review of research ethics committees and informed consent (Trial #2012/546).

### 2.2. DISCovery Method

The *DISCovery* method consisted of three successive steps: (1) psychosocial risk diagnosis; (2) development of interventions; and (3) implementation of the interventions. In the first step, we used baseline survey results to assess the (lack of) balance between key elements of the DISC-R Model (i.e., job demands, resources, and detachment from work) in combination with health, well-being, and performance outcomes for each participating unit. We used internal benchmarks and external reference groups to determine whether scores were relatively high or low. An example of an assumed lack of balance is the combination of (relatively) high scores on emotional demands, low scores on emotional resources and/or emotional detachment, and high scores on emotional exhaustion. In the second step of the method (i.e., intervention development), we used a six-step PAR approach to raise support and ownership among employees and management for identified risks and corresponding—yet to be developed—interventions.

First, we communicated outcomes of the psychosocial risk diagnosis to line management and human resources advisors (i.e., project group) and to higher management (i.e., steering group). During these feedback meetings, participating units were divided into intervention and comparison groups based on several selection criteria: actual presence of risk scores, response rate (>60%), group size (larger groups were preferred because of possible attrition), management preferences (e.g., units with long-term issues or high absenteeism rates), feasibility (e.g., possible interference of organizational activities), and willingness of the unit to participate. All criteria were rated and discussed during project group and steering group meetings. The final designation of the units to the study conditions was proposed by the researchers and approved by the steering group. Additionally, preliminary intervention ideas were discussed, as well as boundaries to interventions due to feasibility and the project’s scope. For instance, hiring more staff or changing labor conditions was not possible. Second, we presented unit-specific baseline results to the employees during feedback meetings with each intervention unit. The comparison groups received written feedback reports about their baseline results. Also, they were told that if the method would prove to be effective, it could also be applied within their units after completion of this study. Third, subsequent to the feedback meetings, we held brainstorm sessions with each intervention unit about possible work-oriented interventions. Employees were asked for their reactions and ideas regarding the baseline results and informed about the boundaries to possible interventions. After that, we chose interventions with an efficacious and democratic prioritization method [11], resulting in a top-3 of intervention themes for each group. Fourth, we consulted the steering and project group about the top-3 intervention lists and possible actions for intervention implementation. Concerns of all stakeholders regarding urgency and feasibility were taken into consideration in determining group-specific action plans. Note that, as a consequence of this specific step, the action plans did not necessarily address all top-3 intervention themes to the same extent. Fifth, we reported the action plans to the intervention units and asked for their reactions and commitment, leaving participants a certain period of time (e.g., 1–2 weeks) to react or provide input to the action plans [13]. Ultimately, higher management decided which unit-specific interventions would be implemented in close consultation with the employees, lower management, and researchers. In the third and final step of the method, unit-specific interventions were further developed and implemented (see Appendix A for more detailed information about the interventions), with support of internal and external subject matter experts when necessary. The interventions were mainly targeted at increasing job resources and/or recovery that match with specific types of job demands (i.e., cognitive, emotional, or physical), depending on the unique unit profiles. In sum, based on outcomes of the PAR-approach, the exact unit-specific intervention programs were determined. Table 2 provides an overview of the results of the successive steps of the *DISCovery* method for each intervention group.

### 2.3. Variables and Instruments

Key variables with regard to the psychosocial risk diagnosis were job demands, job resources, and recovery on the one hand (i.e., proximal variables), and health, well-being, and performance outcomes on the other (i.e., distal variables). After consultation with the project and steering group, we assessed some additional, central work-related characteristics, including work break conditions, recovery during work, teamwork, work-home and home-work interference (i.e., proximal variables). Effects were evaluated with intervention-specific target variables that were selected for each intervention group individually, based on their intervention program. Note that target variables were not an exact reflection of the unit-specific psychosocial risk diagnosis, but of the outcomes of the entire *DISCovery* method. Each variable was measured at each occasion.

#### 2.3.1. Proximal Variables: Work-Related Characteristics

Cognitive, emotional, and physical job demands and job resources were measured with the shortened DISC Questionnaire 3.0 (DISQ-S 3.0 Eindhoven University of Technology, Eindhoven, The Netherlands) [26]. Previous versions of this international and widely used questionnaire have demonstrated good psychometric properties [15,27]. Each DISQ-scale consists of three items, except for the cognitive job resources scales, which has one additional item due to less psychometric properties in the past. All items were rated on a 5-point frequency scale ranging from 1 (never or very rarely) to 5 (very often or always). Examples of the items for job demands are “I need to display high levels of concentration and precision at work” (cognitive; Cronbach’s *α* = 0.66), “I have to do a lot of emotionally draining work” (emotional; Cronbach’s *α* = 0.77), and “I have to perform a lot of physically strenuous tasks to carry out my job” (physical; Cronbach’s *α* = 0.82). Example items of job resources are “I have the opportunity to determine my own work method” (cognitive; Cronbach’s *α* = 0.55), “I receive emotional support from others (e.g., clients, colleagues, or supervisors) when a threatening situation at work occurs” (emotional; Cronbach’s *α* = 0.88), and “I am able to use adequate technical equipment to accomplish physically strenuous tasks” (physical; Cronbach’s *α* = 0.72).

We measured off-job recovery using the DISQ-R, an internationally used scale developed by De Jonge and colleagues [15]. This scale consists of a cognitive, emotional, and physical component of detachment after work. Each component was measured with three items, which were rated on a 5-point frequency scale ranging from 1 (never) to 5 (always). Examples items are “After work, I put all thoughts of work aside” (cognitive; Cronbach’s *α* = 0.75), “After work, I emotionally distance myself from work” (emotional; Cronbach’s *α* = 0.76), and “After work, I shake off the physical exertion from work” (physical; Cronbach’s *α* = 0.66). 

Work break conditions were measured with three items based on commonly used items for assessing the quality, duration, and number of work breaks [28]. An example item is “The quality of my work breaks is good”. Each item was rated on a 5-point Likert scale ranging from 1 (strongly disagree) to 5 (strongly agree). Cronbach’s alpha was 0.90.

Recovery during work was measured with three items reflecting the three DISC-R dimensions [15]: “During a work break, I think of things other than work” (cognitive), “During a work break, I emotionally distance myself from work” (emotional), and “During a work break, I shake off the physical exertion from work” (physical). Items were rated on a 5-point frequency scale ranging from 1 (never or very rarely) to 5 (very often or always). Cronbach’s alpha for this scale was 0.88.

Teamwork was measured with a scale consisting of three items of the COMPaZ [29], a well-validated Dutch version of the Hospital Survey on Patient Safety Culture of the Agency for Healthcare Research and Quality [30]. An example item is “When a lot of work needs to be done quickly, we work together as a team to get the work done”. The items were rated on a 5-point Likert scale ranging from 1 (strongly disagree) to 5 (strongly agree). Cronbach’s alpha for this scale was 0.86.

#### 2.3.2. Distal Variables: Health, Well-Being, and Performance Outcomes

Concentration problems were measured with four items derived from a semantic differential scale developed by Meijman [31]. The items were rated on a 5-point response scale with two extremes, for example “No concentration difficulties” vs. “Concentration difficulties” and “No difficulties paying attention” vs. “Attention keeps fading”. Cronbach’s alpha for this scale was 0.94.

Emotional exhaustion was measured with the well-validated Dutch version of the Maslach Burnout Inventory [32]. The scale consisted of five items (e.g., “I feel emotionally drained from my work”), which were rated on a 7-point frequency scale ranging from 0 (never) to 6 (always). Internal consistency reliability (Cronbach’s alpha) of this scale was 0.87.

Work satisfaction can be viewed as a one-dimensional and general construct, resulting from positive and negative work experiences [33]. It was measured with one item: “I am satisfied with my present job”. This item was rated on a 5-point Likert scale ranging from 1 (strongly disagree) to 5 (strongly agree).

Individual and team work performance were assessed by asking the respondents to rate their own work performance and the work performance of their team separately on a 10-point scale ranging from 1 (very bad) to 10 (very good) cf. [34].

### 2.4. Comparability of Intervention and Comparison Groups

Because participants could not be randomly allocated to intervention and comparison groups, we tested if subsamples differed in gender, age, education, marital status, and working hours at Time 1 (see Table 1) by calculating *t-*tests and chi-square difference tests. Previous research has shown that these demographic characteristics are associated with health, well-being, and performance outcomes [35,36]. There were significant age differences between the intervention and comparison groups in the nursing department (*p* < 0.05). The laboratory subsamples differed on marital status and working hours (*p* < 0.001 and *p* < 0.01, respectively). Hence, these variables were included as control variables in the respective case-specific analyses.

With regard to the predictor and outcome variables, we found several statistical differences between the intervention and comparison groups. For instance, the intervention groups scored significantly lower on various types of job resources (Cases 1 and 2) and detachment from work (Case 1) than their respective comparison group. However, as intervention groups were mainly selected based on the presence of risk scores, these differences can be viewed as an inevitable result of the selection procedure. Moreover, they reaffirm the suitability of the selection of these specific intervention groups with respect to the overarching goal of optimizing job resources and recovery.

### 2.5. Statistical Analysis

We evaluated effects of the interventions by performing multilevel regression analyses with MLwiN 2.25 [37]. This technique has several advantages compared to standard methods for analyzing longitudinal data (e.g., repeated-measures analysis of variance), such as the inclusion of cases with incomplete data and less restrictive missing data assumptions [38]. In the current study, data can be distinguished at two levels: measurement occasions (Level 1) nested within persons (Level 2). The multilevel models for both Case 1 (i.e., nursing department) and Case 2 (i.e., laboratory) included the intervention group and the Time 2 and Time 3 measurements as dummy variables. As such, the reference categories in these multilevel models are the case-specific comparison group and Time 1. The intercept represents the expected overall outcome at Time 1 for the department (i.e., intervention and comparison group), whereas Time 2 and Time 3 refer to the overall outcome at the two follow-up measurements, respectively. The intervention group variable refers to the difference between intervention and comparison group at Time 1. Additionally, interactions between the intervention group with Time 2 and Time 3 were modeled, reflecting between-group differences in model trajectories over time. Finally, as mentioned earlier, case-specific control variables were included in the analyses.

## 3. Results

We will discuss the most important quantitative results for each case; that is, the significant between-group differences in model trajectories over time (i.e., interaction effects). These results reveal on which variables members of the intervention group showed different score patterns over time than members of its comparison group. A complete overview of all baseline means and comparison between groups can be found in Appendix B, whereas the results of the multilevel analyses are reported in Appendix C.

### 3.1. Case 1: Nursing Department

Group means of the intervention and comparison group within the nursing department for each of the target variables at each measurement occasion are shown in Table 3, as well as the variance components of the variables. The variance associated with persons (i.e., individual differences) ranged from 24% to 66%, whereas the remaining variance (34–76%) was associated with measurement occasions (i.e., within-person differences). Thus, overall, considerable proportions of variance in the target variables could be attributed to within-person fluctuations over time.

Multilevel models showed significant positive interaction effects for the intervention group at Time 2 for these variables, implying a positive change between Time 1 and Time 2 in emotional resources (*β* = 0.18; *p* < 0.05), physical resources (*β* = 0.20; *p* < 0.05), and cognitive detachment (*β* = 0.24; *p* < 0.01) for members of the intervention group relative to the change trajectories for members of the comparison group. A similar effect was found for work break conditions at Time 2 (*β* = 0.23; *p* < 0.01). These findings provide support for our first hypothesis H1 (proximal outcomes). Furthermore, the models showed a significant negative interaction effect for members of the intervention group at Time 3 for concentration problems (*β* = −0.20; *p* < 0.05), implying a decrease in concentration problems between Time 1 and Time 3 relative to the change trajectory for members of the comparison group. This finding provides support for our second hypothesis H2 (distal outcomes). Table 4 gives an overview of the significant interaction effects of this case. No significant interaction effects were found for the remaining target variables.

### 3.2. Case 2: Laboratory

Table 5 shows the group means of the intervention and comparison group within the laboratory department for each of the target variables at each measurement occasion, together with the corresponding variance components. Variance associated with persons ranged from 32% to 77%, whereas 24–68% of the variance was associated with measurement occasions.

At Time 2, significant positive interaction effects were found for the proximal outcomes (H1) emotional resources (*β* = 0.26; *p* < 0.01) and teamwork (*β* = 0.22; *p* < 0.05) as well as for the distal outcomes (H2) work satisfaction (*β* = 0.56; *p* < 0.001) and team performance (*β* = 0.29; *p* < 0.05), indicating positive changes in the scores on these variables between Time 1 and Time 2 for members of the intervention group relative to the change trajectories for members of the comparison group. The positive changes in emotional resources and team performance for members of the intervention group extended to the next measurement occasion, as positive interaction effects were also found for these variables at Time 3 (*β* = 0.26; *p* < 0.01, and *β* = 0.32; *p* < 0.05, respectively). See Table 4 for an overview of the significant interaction effects within this case. There were no significant interaction effects for the remaining target variables. For emotional exhaustion, this might be due to the relatively high within-person stability, with only 23% of the variance being associated with differences within persons (i.e., occasion level).

## 4. Discussion

The aim of this quasi-experimental intervention study was to quantitatively assess the effectiveness of the *DISCovery* method. The overall purpose was to influence the range of target variables for all cases. However, for every group the actual focus differed, depending on their group-specific psychosocial risk profile and interventions. We will first shortly discuss the results of each separate case, before moving on to a general discussion.

In Case 1 (i.e., nursing department), the focus of the intervention program was on increasing (emotional and physical) job resources, enhancing recovery, and decreasing concentration problems. Indeed, relative to members of the comparison group, positive changes were visible throughout the intervention program regarding emotional and physical job resources and concentration problems, which were all part of the initial risk profile of the intervention group. In addition, similar changes were visible for cognitive detachment and work break conditions. Most positive changes were visible in the first year of the study (Time 1–Time 2), such as increases in job resources and cognitive detachment, as well as improved work break conditions shortly after the implementation of work breaks. The lean management intervention in the second year progressed somewhat slower than planned, mainly because higher hospital management expressed their wish to first align this intervention with other ongoing lean management activities within the organization. As such, it is plausible that Time 3 measurements followed too soon to capture possible benefits of this intervention. Moreover, the decrease in concentration problems spreads over the entire intervention period (Time 1–Time 3), which could indicate a joint result of the different interventions that were implemented throughout the study. Finally, the participation rate of the job crafting intervention was not high (50%), mainly caused by the fact that the workshops were organized outside regular working hours, which often interfered with private life. Even though positive changes were visible for the targeted outcomes of this particular intervention (i.e., job resources and recovery), reaching the entire target population might have yielded more and/or stronger effects.

In Case 2 (i.e., laboratory), the group-specific psychosocial risk profile consisted of low cognitive, emotional and physical job resources, low physical detachment, high emotional exhaustion, and low work satisfaction and team performance. As a result of the *DISCovery* method, however, the focus of the intervention program was mainly on social and emotional work aspects (e.g., communication, teamwork). Positive changes that were found for members of this group were very much in line with this specific focus, that is, throughout the intervention program there was an increase in emotional resources, work satisfaction, team performance, and teamwork, relative to the comparison group. Similar to Case 1, most positive changes for members of this group were visible between Time 1 and Time 2. As the participatory approach to intervention development took place between Time 1 and Time 2 (i.e., *DISCovery* method, step 2), it seems that the participation itself may have had an additive positive effect on the way people perceived their work situation [23]. However, the longer-term positive changes for emotional job resources and team performance do indicate a positive effect of the entire intervention program on the work situation, in particular with respect to communication and cooperation. 

The average effect size in this study was 0.27 (ranging from 0.18 to 0.56), which is satisfactory and in accordance with the effect sizes of most multi-modal stress management interventions [39]. Taking all outcomes into account, specific positive changes in the work situation were found for the intervention groups, which were in line with the specific intervention programs. However, it is important to realize that looking at changes over time for the intervention groups relative to the comparison group implies a certain dependency between the group results. For example, the fact that we found most positive effects for the intervention groups within these cases between Time 1 and Time 2 is not only due to positive changes in the intervention groups, but also to negative changes in the comparison groups in the same period. Process evaluation pointed out that this was a period in which employees were confronted with organizational changes and a flu epidemic, and where, consequently, upward trends in job insecurity and/or workload were reported. Then again, the fact that the scores of the intervention group did not reveal these trends (contrary to the comparison groups), may indicate a positive non-specific ‘vaccination’ effect of the interventions, with the groups that received ‘treatment’ being more resistant to negative external influences.

In none of the cases we found effects for cognitive resources. This was against our expectations, particularly for the laboratory (Case 2), but interesting though. Cognitive resources refer to the opportunity to determine a variety of task aspects and to use problem-solving skills [15], which are elements that were part of the intervention program (i.e., team workshops) and the overall participatory approach towards the development and implementation of the interventions. A possible reason for these null-findings could be that we assessed changes in cognitive resources at the individual level, whereas intervention development and implementation took place at the team level. As a consequence, cognitive resources might be enhanced at the team level in terms of participative decision-making rather than at the individual level (e.g., individual job control) see also [33]. It would be recommendable to further investigate this aspect in future research.

### 4.1. Theoretical and Practical Implications

First, results underscore the value of both DISC-R and PAR principles for development and implementation of work stress and performance interventions. Using group-specific DISC-R risk profiles as a starting point for idea generation regarding interventions, involving employees and management in the development and implementation of interventions, and tailoring interventions to target groups were all highly-valued elements among the study participants that seemed to have contributed to the success of the intervention programs. With regard to DISC-R theory, empirical findings in this study provide support for the idea that counterbalancing high job demands with job resources and recovery can lead to positive changes in employee health, well-being, and performance outcomes. However, no strong conclusions can be drawn regarding the assumption that job resources and recovery are most effective if they correspond with particular types of job demands (cognitive, emotional, or physical). In the current study, we used the matching principle as a heuristic for the development of the interventions and combined it with the outcomes of the PAR approach. This is in line with De Jonge and Dormann [6], who argued that interventions should address work characteristics that are theoretically known to be related to the desired outcome variables, and with Griffiths [40], who advocated that interventions should take place with the participation and experience of the subjects under study. Results of this study reinforce these notions, indicating a strong practical value of the DISC-R Model and lending support for the effectiveness of the *DISCovery* method.

Second, exposure of employees to the interventions relied greatly on the extent to which interventions received organizational support. A possible way to increase organizational support, besides creating explicit internal support networks, is formalizing active engagement with the intervention content. This can be done, for example, through the incorporation of intervention themes in the agenda of formal team meetings and annual performance appraisals, or by setting up specific employee working groups. Third, to reinforce participation of employees in interventions, intervention activities are best organized during regular work time, thus, requiring organizational facilitation and support. 

Finally, the current study pointed out the pivotal role of the departmental management throughout the entire implementation process, for instance, with respect to recruiting and enthusing employees, as well as managing changes in the work situation [41]. Therefore, it is highly recommendable to pay specific attention to the managerial key figures and offer individual coaching whenever possible to remedy potential implementation issues and strengthen the success of the interventions.

### 4.2. Strengths and Limitations

Although this study has several strengths, such as a participatory action research approach and a strong theoretical embeddedness, a limitation of this study is that the sample sizes are relatively small, which can cause power problems in statistical analyses (i.e., not detecting differences that do exist). This is an issue common to longitudinal intervention studies, caused by the fact that intervention studies are often most meaningfully implemented at local organizational levels [42]. In this study, tailoring interventions to relatively small organizational units inevitably called for unit-level analysis for each separate case. Moreover, as in most longitudinal studies, part of the data was missing due to panel attrition. We dealt with this issue by analyzing our data with multilevel regression analysis, which uses all available data instead of listwise deleting cases with missing data [38].

In addition, Cronbach’s alphas for a few measurement scales were relatively low according to conventional guidelines (>0.70), which may also be (partly) due to the small sample sizes. Particularly the cognitive resources scale yielded a low Cronbach’s alpha (0.55). As removing one of the items from the scale did not substantially improve the reliability or change the results, we decided to adhere to the original, well-validated scale. In previous studies, internal consistency of this scale has shown satisfactory to good results e.g., [21,43]. It could be that the current scale is rather multidimensional in nature, by representing an autonomy aspect as well as an informative aspect of cognitive resources. According to Schmitt [44], the alpha coefficient is not suitable for multidimensional measures, as the use of alpha—as an estimate of reliability—is based on the notion that the measures involved are unidimensional. For future studies, it would be recommendable to increase the number of items related to both types of cognitive resources and to reassess the psychometric properties of this scale.

Inherent to the particular procedure for assigning units to the experimental condition, intervention and comparison groups were non-equivalent at baseline with respect to predictor and outcome variables. A possible consequence is that improvements in the intervention groups and negative changes over time in the comparison groups might be (partly) due to regression towards the mean, as opposed to “(non-)treatment” [23]. If this were true, however, we would expect to observe the trend of regression to the mean for the work-related aspects that were directly addressed by the interventions (e.g., Case 2: emotional resources) as well as for the ones that were not (e.g., Case 2: physical resources). The absence of the latter strengthens the idea that effects can be attributed to the intervention condition rather than natural fluctuations around the mean. In addition, regression to the mean would imply that particularly low or high initial scores (i.e., risk scores at Time 1) are due to random factors (i.e., measurement error), rather than a reflection of the ‘true’ scores of the underlying concept. However, in the PAR procedure we checked if initial risk scores were recognized and acknowledged by management and employees. This enabled us to confirm the representativeness of the scores for the true work situation. As such, we minimized the chance of regression to the mean.

### 4.3. Future Research

An avenue for future research could be the investigation of the optimal timeframe and method for effect evaluation of tailor-made intervention programs. As became clear in the current study, tailored interventions require analysis on the level of the target group, looking at target variables and specific time frames. For instance, for some interventions it takes time to bear fruit, whereas others seem to yield results relatively quickly. Furthermore, as mentioned earlier, effects in distal outcomes (e.g., improved health) may take longer to unfold than proximal effects (e.g., changed work-related characteristics). As a consequence, both short and long time frames may not capture certain effects [25]. Additionally, evaluating effects of tailored interventions may require surveys that are also tailored to a certain extent, capturing the local context and using the daily language of the individuals under study [45]. As such, the challenge would be to integrate different timeframes and the local context into a hybrid, effective, yet efficient method. Another interesting avenue for further research is the inclusion of different kinds of biomarkers which can act as mediators of the associations between our work stress indicators and health and performance outcomes [46]. Finally, results in this study were based on a specific sample and the method that was used to create tailored interventions inherently challenges generalizability and replicability of the results. That is, interventions were developed within the unique context of the respective cases and tailored to the specific needs of participating employees and management. For future research it would be interesting to investigate the practical value of the *DISCovery* method in sectors and organizations other than health care. We expect that the effectiveness of the method does not depend on the type of work, as it is aimed at tailoring interventions to any target group. In other words, it can be seen as a generic approach that becomes more specific and targeted depending on the input of the individuals under study.

## 5. Conclusions

Taken together, results were in line with the overall expectations; that is, positive changes were found for members of the intervention groups relative to the members of the corresponding comparison groups with respect to targeted work-related characteristics (H1; proximal outcomes) and targeted health, well-being, and performance outcomes (H2; distal outcomes). In all cases, effects for targeted proximal outcomes were already visible at the first follow-up measurements, whereas in two out of three cases effects on distal outcomes were visible only at the second follow-up measurements. This is in line with the idea that proximal outcomes are more sensitive to the intervention process and may take less time to unfold than distal outcomes [24].

This study indicates that the *DISCovery* method is an effective, promising and systematic approach for optimizing psychosocial working conditions and improving health, well-being, and performance of employees in health care. As such, it provides an important contribution to bridging the gap between theoretical knowledge regarding work stress prevention and corresponding practical solutions.

## Figures and Tables

**Table 1:** Baseline demographic characteristics and response rates of intervention and comparison groups (*N* = 111).

	Nursing Department		Laboratory	
	IG	CG	IG	CG
*Gender*				
Male	7.1%	9.4%	11.8%	26.3%
Female	92.9%	90.6%	88.2%	73.7%
*Age*				
Mean years (SD)	40.4 (10.2)	34.1 (10.8)	48.6 (11.4)	45.5 (10.5)
*Education*				
High school	28.6%	25.0%	11.8%	5.3%
Vocational education	21.4%	43.8%	29.4%	36.8%
Higher education	50.0%	31.3%	58.8%	57.9%
*Marital status*				
Single	32.1%	22.6%	70.6%	10.5%
Cohabiting/Married	67.9%	77.4%	29.4%	89.5%
*Irregular working hours*				
Yes, including night shifts	78.6%	90.6%	17.6%	89.5%
Yes, excluding night shifts	3.6%	0.0%	58.8%	10.5%
No	17.9%	9.4%	23.5%	0.0%
*Response rates*				
Time 1	*N* = 28 (90%)	*N* = 32 (86%)	*N* = 17 (74%)	*N* = 18 (95%)
Time 2	*N* = 19 (61%)	*N* = 26 (70%)	*N* = 17 (77%)	*N* = 17 (89%)
Time 3	*N* = 26 (84%)	*N* = 35 (95%)	*N* = 15 (75%)	*N* = 17 (100%)

Note: IG = intervention group; CG = comparison group; SD = standard deviation.

**Table 2:** Results of successive steps of the DISCovery method for each intervention group together with the intervention-specific target variables.

Intervention Group	Step 1: Psychosocial DISC-R Risk Profile	Step 2: Outcomes PAR	Step 3: Intervention Program	Main Target Variables
Nursing Department	High emotional job demands Low emotional and physical job resources High concentration problems	Inefficient work processes, no work breaks at subunit Inefficient cooperation and communication Inadequate physical work space and materials	Implementation of work breaks at subunit Job crafting Lean management Coaching supervisor and working group lean management	General:
				Job resources Detachment Work performance Work satisfaction
				Group-specific:
				Recovery during work Work break conditions Concentration problems
Laboratory	High cognitive job demands Low cognitive, emotional, and physical job resources Low physical detachment Low work satisfaction Low team performance High emotional exhaustion	Dysfunctional cooperation Dysfunctional communication Poor physical work climate	Analysis of departmental cooperation and communication goals Team workshops “Cooperation and Communication” Follow-up workshops Coaching supervisor	General:
				Job resources Detachment Work performance Work satisfaction
				Group-specific:
				Teamwork Emotional exhaustion

**Table 3:** Group means and variance components of the target variables for the intervention group and the comparison group in the nursing department (*N* = 60 at Time 1).

	Nursing Intervention Group			Nursing Comparison Group			Variance (%)	
Variable	T1	T2	T3	T1	T2	T3	Person	Occasion
*General proximal target variables*								
Cognitive resources	3.15	3.12	2.98	3.37	3.12	3.35	50.0	50.0
Emotional resources	3.88	3.93	4.14	4.23	3.85	4.20	57.0	43.0
Physical resources	3.11	3.24	3.27	3.56	3.25	3.53	53.5	46.5
Cognitive detachment	3.88	3.98	3.97	4.16	3.99	3.95	66.1	33.9
Emotional detachment	3.71	3.89	3.87	3.86	3.82	3.76	44.2	55.8
Physical detachment	3.52	3.56	3.72	3.53	3.55	3.58	39.5	60.5
*General distal target variables*								
Work satisfaction	3.79	4.00	3.84	3.88	3.85	3.86	23.9	76.1
Individual work performance	7.70	7.68	7.77	7.70	7.73	7.80	45.7	54.3
Work performance team	7.36	7.58	7.65	7.75	7.76	7.74	38.6	61.4
*Group-specific target variables*								
Concentration problems (D)	2.25	2.19	1.96	1.97	2.11	2.30	60.1	39.9
Recovery during work (P)	3.23	3.05	3.33	2.88	2.78	3.20	38.6	61.4
Work break conditions (P)	3.30	3.63	3.47	3.16	2.81	3.21	63.4	36.6

Note: P = proximal variable; D = distal variable; T1 = Time 1; T2 = Time 2; T3 = Time 3.

**Table 4:** Overview of the significant multilevel results for the target variables within the participating departments.

	Target Variables	Occasion(s)	Effect Size (*β*)
*Case 1: Nursing Department*			*Interactions (Group X Time)*
Proximal outcomes	Emotional resources	T2	0.18 *
	Physical resources	T2	0.20 *
	Cognitive detachment	T2	0.24 **
	Work break conditions	T2	0.23 **
Distal outcomes	Concentration problems	T3	−0.20 *
*Case 2: Laboratory*			
Proximal outcomes	Emotional resources	T2/T3	0.26 **/0.26 **
	Teamwork	T2	0.22 *
Distal outcomes	Work satisfaction	T2	0.56 ***
	Team performance	T2/T3	0.29 */0.32 *

Note: * *p* < 0.05; ** *p* < 0.01; *** *p* < 0.001: Significant higher or lower scores, with the comparison group and Time 1 as reference categories.

**Table 5:** Group means of the target variables for the intervention group and the comparison group in the laboratory (*N* = 35 at Time 1).

	Laboratory Intervention Group			Laboratory Comparison Group			Variance (%)	
Variable	T1	T2	T3	T1	T2	T3	Person	Occasion
*General proximal target variables*								
Cognitive resources	2.80	2.78	2.63	3.22	3.25	3.18	52.0	48.0
Emotional resources	3.29	3.49	3.74	4.20	3.94	4.02	67.4	32.6
Physical resources	2.73	2.78	2.62	3.45	3.25	3.33	38.8	61.2
Cognitive detachment	3.75	3.75	3.76	3.60	3.71	3.78	55.5	44.5
Emotional detachment	3.41	3.45	3.57	3.39	3.45	3.48	53.8	46.2
Physical detachment	3.16	3.43	3.17	3.61	3.75	3.65	55.3	44.7
*General distal target variables*								
Work satisfaction	3.38	3.65	3.57	3.95	3.24	3.89	37.1	62.9
Individual work performance	7.82	7.41	7.50	7.53	7.47	7.56	41.5	58.5
Work performance team	6.00	6.94	6.86	7.68	7.75	7.67	32.4	67.6
*Group-specific target variables*								
Teamwork (P)	3.00	3.40	3.17	4.18	4.10	4.35	57.2	42.8
Emotional exhaustion (D)	2.91	2.79	2.99	2.52	2.44	2.45	76.5	23.5

Note: P = proximal variable; D = distal variable; T1 = Time 1; T2 = Time 2; T3 = Time 3.

## Appendix A

**Table A1:** Description of interventions.

Intervention Group	Intervention	Description
Nursing	1. Work breaks	Formal daily 30-min work breaks were implemented at the daycare subunit of this department, conform existing practices in the rest of the department.
	2. Job crafting	A job crafting intervention was implemented to increase job resources and enhance recovery from work [47]. Based on an existing framework for job crafting training [48], the intervention consisted of the following three steps: (1) An initial workshop, in which participants learned about the basic principles of job crafting and set personal job crafting goals; (2) A 4-week period during which participants worked on reaching their personal job crafting goals and received a weekly e-mail as a reminder of their goals; (3) A final reflection meeting, in which participants reflected upon their goals, shared tips and experiences with each other, and decided what to keep working on in the future.
	3. Lean management	Five employees of the department volunteered to be part of a working group lean management [48]. The task of this group was to initiate lean management within the department and to involve other team members in this process. Supported by the researchers, departmental management, and an internal lean management expert, the group selected and implemented several lean management tools (i.e., 5S, kaizen, idea board). Monthly meetings were organized to discuss the overall progress, difficulties, and next action steps with the working group. Additional support meetings were planned when necessary.
	4. Coaching trajectories	An external coach provided individual coaching to the direct supervisor of the intervention group and joint coaching sessions to the working group lean management.
Laboratory	1. Analysis departmental goals	An in depth analysis of departmental goals and ambitions (specifically regarding communication and cooperation) was carried out, using a supplementary tailored questionnaire. The results of this analysis were presented to the departmental management and used as input for the team workshops.
	2. Team workshops	A first round of team workshops about goals, communication, and cooperation was organized for the whole team, including the departmental management. During these workshops, small working groups were initiated to deal with specific problems (e.g., physical work climate). In a series of follow-up team workshops, participants reflected upon the progress of both the working groups and the team in general, with respect to communication and cooperation.
	3. Coaching trajectory	An external coach provided individual coaching to the direct supervisor of the intervention group.

## Appendix B

**Table A2:** Baseline means and standard deviations of study variables for all participating groups.

Variables	Nursing				Laboratory			
	IG		CG		IG		CG	
	M	SD	M	SD	M	SD	M	SD
Cognitive demands	4.43	0.46	4.35	0.33	4.18	0.39	4.33	0.46
Emotional demands	3.21 *	0.46	2.84 *	0.70	2.59	0.59	2.49	0.45
Physical demands	3.18 *	0.90	3.94 *	0.65	3.02	1.01	2.61	0.37
Cognitive resources	3.15	0.58	3.37	0.44	2.80 *	0.57	3.21 *	0.53
Emotional resources	3.88 *	0.51	4.23 *	0.67	3.29 ***	0.73	4.20 ***	0.56
Physical resources	3.11 *	0.63	3.56 *	0.65	2.73 **	0.64	3.45 **	0.70
Cognitive detachment	3.88 **	0.38	4.16 **	0.40	3.75	0.65	3.60	3.61
Emotional detachment	3.71	0.48	3.86	0.54	3.41	0.52	3.39	0.55
Physical detachment	3.52	0.55	3.53	0.65	3.16	0.76	3.61	0.60
Recovery during work	3.23	0.80	2.88	0.66	3.35	0.79	3.56	0.70
Concentration problems	2.25	1.03	1.97	0.79	1.71	0.52	2.16	0.83
Emotional exhaustion	2.55	1.07	2.72	0.85	2.91	1.10	2.52	0.96
Work satisfaction	3.79	1.10	3.88	0.70	3.38 *	1.02	3.95 *	0.40
Individual work performance	7.70	0.61	7.70	0.68	7.82	0.53	7.53	0.77
Team performance	7.36	1.25	7.75	0.72	6.00 **	1.59	7.68 **	0.58
Work break conditions	3.30	0.84	3.16	0.71	3.47	0.91	3.79	0.45
Teamwork	4.15	0.46	4.35	0.46	3.00 ***	0.69	4.18 ***	0.39

Note: Means are tested with *t*-tests (horizontal comparisons). The contrast is: “Intervention group (IG)” vs. “Control group (CG)”. * *p* < 0.05; ** *p* < 0.01; *** *p* < 0.001: significantly higher/lower means.

## Appendix C

**Table A3:** Multilevel models for change over time and intervention effects within the nursing department (*N* = 60 at Time 1).

	Outcome Variable											
	Cognitive Resources		Emotional Resources		Physical Resources		Cognitive Detachment		Emotional Detachment		Physical Detachment	
Variable	B	SE	B	SE	B	SE	B	SE	B	SE	B	SE
Intercept	3.36 ***	0.09	4.24 ***	0.11	3.54 ***	0.11	4.12 ***	0.08	3.80 ***	0.09	3.49 ***	0.10
Time and Intervention												
Intervention group	−0.18	0.13	−0.36 *	0.17	−0.36 *	0.17	−0.27 *	0.12	−0.10	0.13	0.00	0.15
Time 2	−0.23 *	0.10	−0.41 **	0.12	−0.30 *	0.12	−0.16 *	0.08	0.05	0.10	0.07	0.13
Time 3	0.00	0.10	−0.07	0.11	−0.01	0.11	−0.10	0.07	−0.13	0.09	0.14	0.12
Intervention Group × T2	0.16	0.15	0.35 *	0.17	0.38 *	0.19	0.31 **	0.12	0.16	0.15	0.01	0.19
Intervention Group × T3	−0.19	0.14	0.30	0.16	0.04	0.17	0.19	0.11	0.16	0.14	0.04	0.17
Control variables												
Age	0.00	0.01	0.00	0.01	0.01	0.01	0.00	0.00	−0.00	0.00	0.00	0.01
Variance components												
Individual	0.14 ***	0.04	0.28 ***	0.06	0.21 ***	0.05	0.15 ***	0.03	0.11 ***	0.03	0.15 ***	0.04
Occasion	0.13 ***	0.02	0.16 ***	0.02	0.18 ***	0.03	0.07 ***	0.01	0.13 ***	0.02	0.21 ***	0.03
	**Work Satisfaction**		**Individual Work Performance**		**Team Performance**		**Concentration Problems**		**Recovery during Work**		**Work Break Conditions**	
Variable	B	SE	B	SE	B	SE	B	SE	B	SE	B	SE
Intercept	3.83 ***	0.15	7.68 ***	0.10	7.76 ***	0.15	1.93 **	0.14	2.88 **	0.12	3.13 **	0.12
Time and Intervention												
Intervention group	−0.05	0.22	−0.05	0.15	−0.39	0.22	0.41	0.22	0.34	0.18	0.19	0.18
Time 2	−0.03	0.21	0.00	0.12	−0.01	0.19	0.09	0.16	−0.08	0.15	−0.30 *	0.12
Time 3	0.04	0.19	0.11	0.11	−0.02	0.18	0.29 *	0.15	0.30 *	0.14	0.08	0.11
Intervention Group × T2	0.24	0.31	0.05	0.18	0.33	0.27	−0.28	0.24	−0.09	0.23	0.51 **	0.19
Intervention Group × T3	0.03	0.28	0.01	0.17	0.27	0.26	−0.50 *	0.22	−0.26	0.21	0.08	0.17
Control variables												
Age	0.00	0.01	0.01	0.01	0.01	0.01	−0.02 *	0.01	0.00	0.01	0.00	0.01
Variance components												
Individual	0.19 *	0.08	0.17 ***	0.04	0.28 **	0.09	0.42 **	0.10	0.17 **	0.06	0.33 **	0.07
Occasion	0.56 ***	0.08	0.19 ***	0.03	0.46 ***	0.07	0.31 **	0.05	0.29 **	0.04	0.19 **	0.03

Note: Time 1 and the comparison group are reference categories.** p* < 0.05; *** p* < 0.01; *** *p* < 0.001.

**Table A4:** Multilevel models for change over time and intervention effects within the laboratory (*N* = 35 at Time 1).

	Outcome Variable											
	Cognitive Resources		Emotional Resources		Physical Resources		Cognitive Detachment		Emotional Detachment		Physical Detachment	
Variable	B	SE	B	SE	B	SE	B	SE	B	SE	B	SE
Intercept	3.31 ***	0.26	4.16 ***	0.41	3.52 ***	0.37	3.33 ***	0.34	3.12 ***	0.32	3.00 ***	0.37
Time and Intervention												
Intervention group	−0.06	0.24	−0.81 *	0.36	−0.65	0.36	0.17	0.30	0.11	0.29	−0.02	0.34
Time 2	−0.01	0.13	−0.30 *	0.13	−0.20	0.21	0.10	0.13	0.04	0.13	0.08	0.15
Time 3	−0.03	0.13	−0.21	0.12	−0.12	0.20	0.19	0.13	0.10	0.12	0.03	0.15
Intervention Group × T2	−0.08	0.19	0.52 **	0.19	0.19	0.30	−0.04	0.19	0.01	0.18	0.26	0.22
Intervention Group × T3	−0.19	0.19	0.51 **	0.19	−0.05	0.30	−0.20	0.19	0.01	0.19	−0.02	0.23
Control variables												
Marital status	−0.31	0.17	−0.06	0.26	−0.06	0.24	0.01	0.22	−0.02	0.21	−0.28	0.24
Irregular shift (excl. Night)	−0.38	0.22	0.07	0.40	−0.14	0.30	0.33	0.28	0.25	0.27	0.43	0.31
Nightshift	−0.02	0.25	−0.04	0.34	−0.06	0.35	0.26	0.33	0.27	0.31	0.67	0.36
Variance components												
Individual	0.08 **	0.03	0.24 **	0.08	0.12	0.06	0.17 **	0.05	0.15 **	0.05	0.20 **	0.07
Occasion	0.15 **	0.03	0.14 ***	0.03	0.34 ***	0.06	0.14 ***	0.03	0.14 ***	0.03	0.20 ***	0.04
	**Work Satisfaction**		**Individual Work Performance**		**Team Performance**		**Teamwork**		**Emotional Exhaustion**			
Variable	B	SE	B	SE	B	SE	B	SE	B	SE		
Intercept	4.60 ***	0.37	7.13 ***	0.35	8.25 ***	0.40	4.48 ***	0.27	3.47 ***	0.59		
Time and Intervention												
Intervention group	−0.72 *	0.34	0.49	0.31	−1.72 ***	0.38	−1.34 ***	0.25	−0.04	0.51		
Time 2	−0.70 ***	0.16	−0.06	0.16	0.08	0.27	−0.09	0.15	−0.02	0.16		
Time 3	−0.08	0.16	0.01	0.16	−0.04	0.26	0.18	0.15	0.02	0.16		
Intervention Group × T2	1.09 ***	0.24	−0.28	0.23	0.82 *	0.39	0.44 *	0.22	−0.17	0.24		
Intervention Group × T3	0.29	0.24	−0.30	0.23	0.91 *	0.39	−0.00	0.22	0.01	0.24		
Control variables												
Marital status	−0.19	0.24	−0.15	0.22	−0.35	0.25	0.05	0.17	0.43	0.37		
Irregular shift (excl. Night)	−0.42	0.30	0.41	0.28	−0.30	0.32	−0.21	0.21	−1.11 *	0.49		
Nightshift	−0.65	0.35	0.41	0.33	−0.56	0.37	−0.32	0.25	−1.02	0.57		
Variance components												
Individual	0.17 **	0.06	0.15 **	0.06	0.05	0.08	0.06	0.03	0.60 ***	0.16		
Occasion	0.23 ***	0.04	0.22 ***	0.04	0.62 ***	0.11	0.20 ***	0.04	0.22 ***	0.04		

Note: Time 1 and the comparison group are reference categories.** p* < 0.05; *** p* < 0.01; *** *p* < 0.001.
